# Supplementary material for: Changes in Cell Morphology and Actin Organization in Embryonic Stem Cells Cultured under Different Conditions
Source: Cells. 2021 Oct 23;10(11):2859. doi: 10.3390/cells10112859 (PMC8616278; doi:10.3390/cells10112859)
Supplement: Supplementary file 1 [file cells-10-02859-s001.zip › cells-1368275-supplementary.pdf]

Supplementary Information:

Changes in cell morphology and actin organization in embryonic stem cells cultured under different conditions

Barooji, Y. F.[1], Hvid, K. G.[1], Isturiz, I.[1], Brickman, J.M.[2], Oddershede, L.B.[1*], Bendix, P. M[1*].

Affiliations:

[1]Niels Bohr Institute, University of Copenhagen, 2100 Copenhagen, Denmark

[2]The Novo Nordisk Foundation Center for Stem Cell Biology, 2200 Copenhagen, Denmark

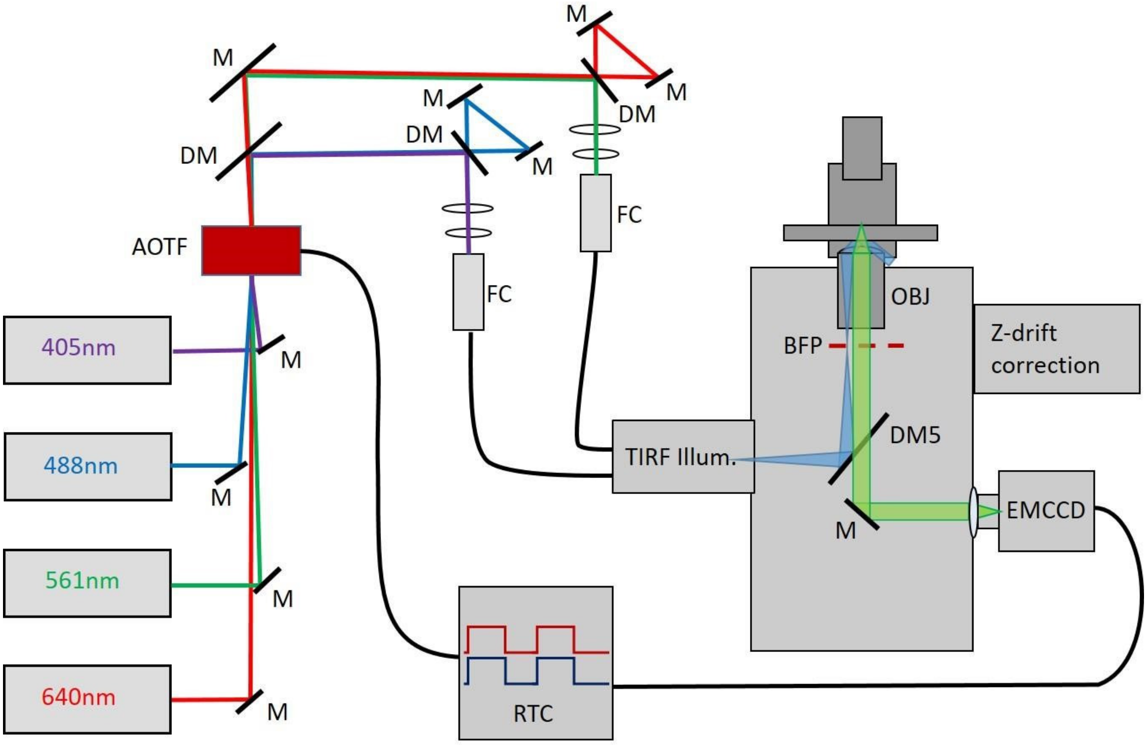

Supplementary Figure S1. Schematic of the super-resolution setup**.** Lasers of wavelengths 405 nm (40 mW, Olympus), 488 nm (200 mW, Cobolt), 561 nm (200 mW, Cobolt), and 640 nm (200 mW, Toptica) are directed by mirrors through the aperture of an acousto-optic tunable filter (AOTF) (AA Opto Electronic). The beams are split by dichroic mirrors (DM) (AHF Analysentechnik), and the 405 and 488 nm beams are coupled into one single-mode optical fiber (FC) (Qioptic); likewise, the 561 and 640 nm beams are coupled into a second optical fiber. The TIRF illuminator allows motorized control of the focus position of the excitation light on the back focal plane (BFP) of the objective (OBJ) (1.45 NA, UAPON OTIRF, Olympus). Drift in the axial direction is continuously corrected by the Z-drift correction module (IX3-ZDC, Olympus). Images are captured on an EMCCD camera (ImagEM X2, Hamamatsu), which is coupled to the real-time controller (RTC, Olympus), that synchronizes the camera shutter with the AOTF in order to minimize sample illumination and bleaching.

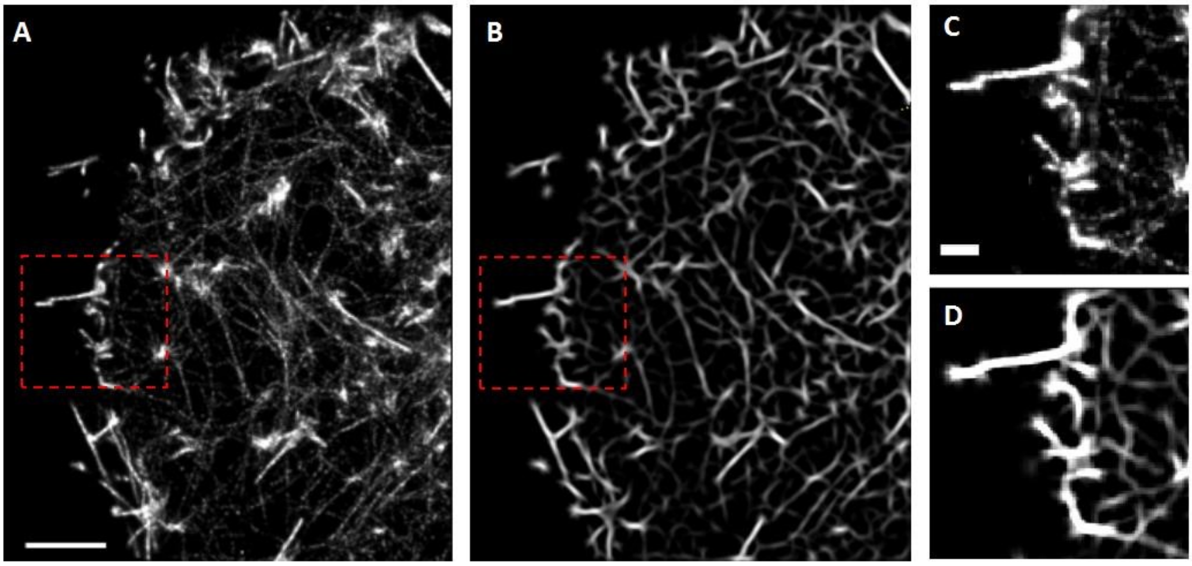

Supplementary Figure S2. Spatial resolution enhancement. (**A**) Reconstructed STORM image of the actin cytoskeleton in a stem cell in 2i media. (**B**) Image corresponding to (A) after 2D Gabor filtering. (**C**–**D**) Magnified views of the red boxed regions in images (A) and (B), respectively. Scale bar shown in (A) is also valid in (B) and is 2 μm. Scale bar shown in (C) is also valid in (D) and is 0.5 μm

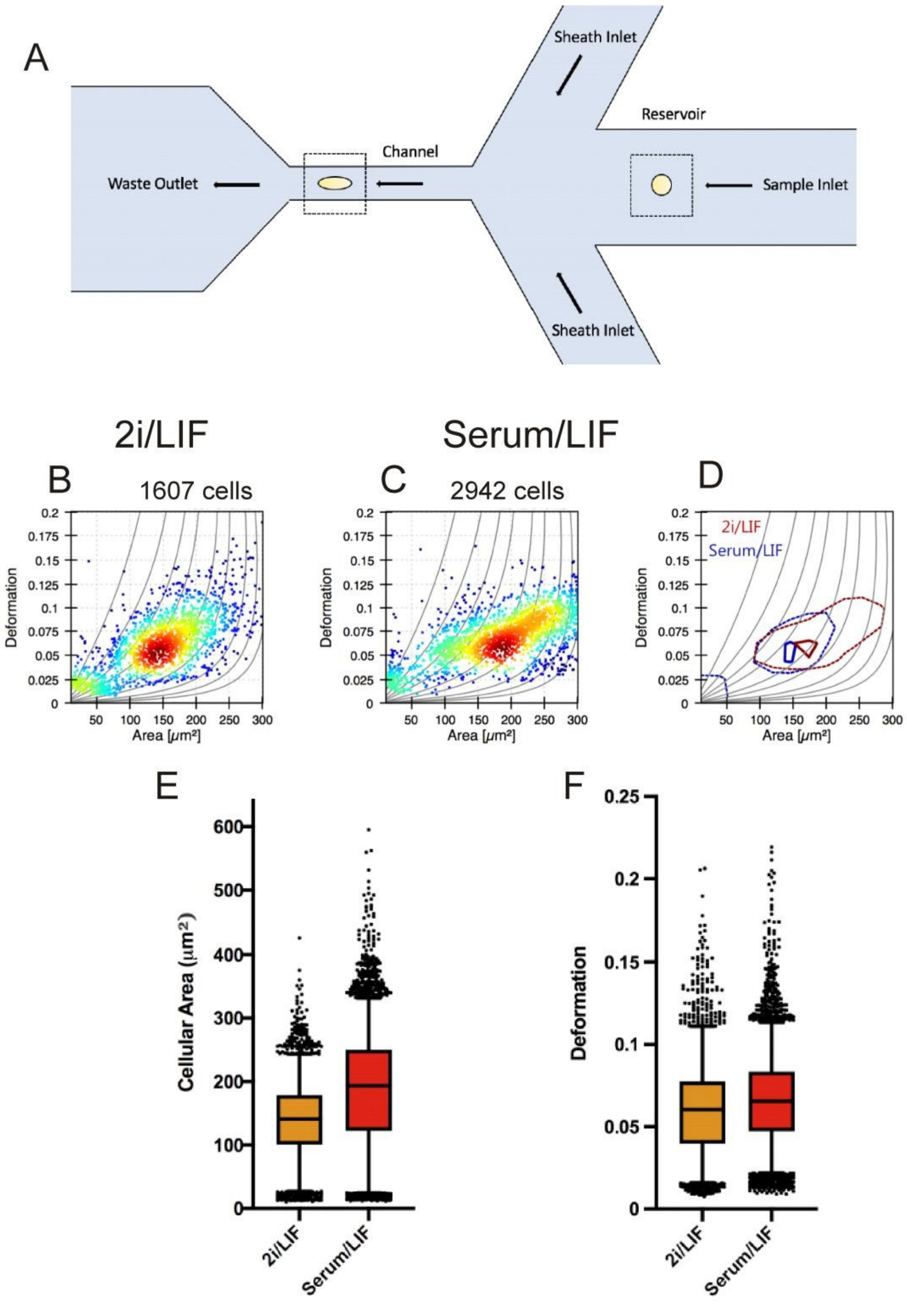

Supplementary Figure S3. Flow cytometry of mESCs cultured in 2i/LIF or Serum/LIF. (**A**) Schematic drawing of our microfluidic flow setup. (**B**) Deformation versus area measured for 1607 cells cultured in 2i/LIF media. (**C**) Deformation versus area for 2942 cells cultured in Serum/LIF media. (**D**) Comparison between the deformations in the two media is assessed by plotting a contour around the central peaks. (**E**) Box plot showing the area of the cells in the two respective media. (**F**) Deformation parameter for the cells cultured in the two respective media.

Real-Time Deformability Cytometry

Deformability cytometry was carried out on a system developed and commercialized by the company ZellMechanik, Dresden, Germany.

The degree of deformation of cells flowing through the narrow channel (see Supplementary Figure 3A) is given as

$$Deformation = 1 - \frac{2\sqrt{\pi\, Area}}{Perimeter}$$

Cells with circular shapes have deformation parameter close to 0 whereas highly deformed cells have values closer to 1.

The data for the two populations of cells show that cells exhibit similar deformation under both conditions, see Supplementary Figure S3.
